# A Case of Blastic Plasmacytoid Dendritic Cell Neoplasm with Unusual Presentation

**DOI:** 10.4274/tjh.galenos.2018.2018.0181

**Published:** 2019-02-07

**Authors:** Sneha Dhariwal, Monica Gupta

**Affiliations:** 1 Delhi State Cancer Institute, Department of Oncopathology, Dilshad Garden, Delhi, India

**Keywords:** Blastic plasmacytoid dendritic cell neoplasm, Testicular involvement, Immunophenotyping

## To the Editor,

Blastic plasmacytoid dendritic cell neoplasm (BPDCN) was categorized in the 2008 World Health Organization classification of hematological diseases under acute myeloid leukemia and related precursor neoplasms [1]; however, in the revised edition 2017, it is a separate entity [2]. BPDCN predominantly affects elderly patients with a mean age between the sixth and seventh decades with a male predilection (M:F=3:1), but it can occur even in children [3,4]. It has an aggressive behavior and rapid systemic dissemination. We herein report a case of BPDCN in an adolescent patient with unusual presentation. 

**Figure 1:**
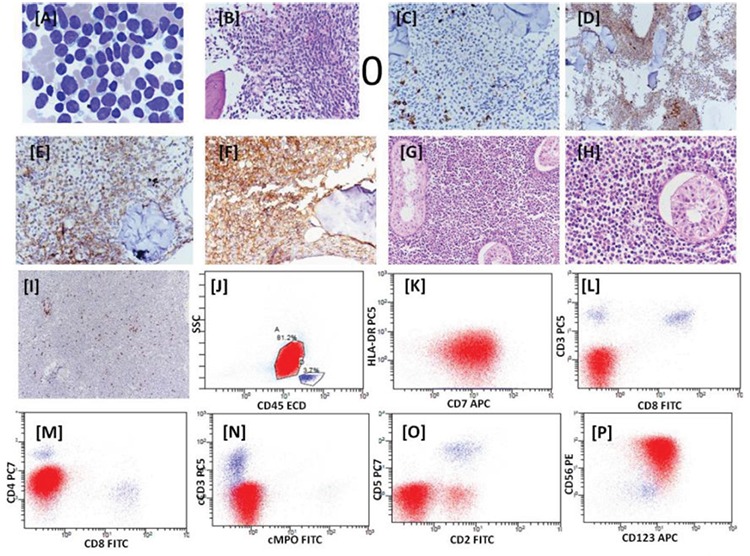
Bone marrow aspiration showing blast-like cells, Giemsa stain, 100x (A). Bone marrow biopsy showing replacement of marrow by monomorphic cells, hematoxylin and eosin stain, 40x (B). Immunohistochemistry on bone marrow biopsy with CD3 (C), CD7 (D), CD4 (E), and CD43 (F). Histology of testicular mass, hematoxylin and eosin stain, 20x (G), and hematoxylin and eosin stain, 40x (H). Immunohistochemistry of testicular mass with CD3 (I). Dot plots with CD45 vs. SSC showing blast population highlighted in red and lymphocytes in blue (J-P). These dot plots demonstrate the expression of CD7_dim_, CD4_dim_, CD56, and CD123. The blasts are negative for CD19, CD10, cCD3, cMPO, CD3, CD8, CD5, CD2, and HLA-DR. 128x96 mm (72x72 DPI).

A 13-year-old male presented with fever and weakness for 10 days. The patient had a history of left testicular swelling for which he had undergone orchidectomy 2 months ago. Routine hematological investigations revealed hemoglobin of 9.1 g/dL, total leukocyte count of 5x103/µL, platelet count of 80x103/µL, and 18% blasts on differential count. Bone marrow aspirate showed 90% blasts with lymphoid morphology (Figure 1A). Flow cytometric immunophenotyping (FCM) analysis of bone marrow revealed 80% blasts with CD45dim and low side scatter. The cells were also positive for CD4, CD56, CD38, CD123, and CD7 and negative for CD19, CD20, CD22, CD3, CD8, CD1a, CD34, CD36, TdT, CD61, CD235a, CD13, CD33, CD14, CD64, CD36, CD117, cMPO, cCD79A, and cCD3 (Figures 1J-1P). Bone marrow biopsy revealed near total replacement by monomorphic cells, which on immunohistochemistry were positive for CD4, CD43, and CD7 and negative for CD3 and CD68 (Figures 1B-1F). Paraffin blocks of the testicular mass were reviewed and revealed a tumor comprising small monomorphic round cells with scanty cytoplasm, round to focally indented nuclei with prominent nuclear membrane, and inconspicuous nucleoli. Few mitotic figures were seen. The seminiferous tubules were enclosed and were being indented by the tumor throughout the tissue section. These cells were positive immunohistochemically for CD4, CD43, CD56, and CD123 and negative for CD3, CD68, and CD8 (Figures 1G-1I). Based on histopathological findings, immunohistochemistry, and FCM analysis, the patient was diagnosed with BPDCN. 

BPDCN closely resembles the precursor of plasmacytoid dendritic cells by immunophenotyping, gene expression profiling, and biologic function [5]. Skin manifestations are the main clinical presentation of typical cases of BPDCN, seen in 64%-100% of adult cases, and the diagnosis is made on skin biopsy [2,6]. Extracutaneous involvement often occurs in the lymph nodes, peripheral blood, bone marrow, skeleton, gastrointestinal tract, central nervous system, lungs, mediastinum, and pancreas [7]. Our patient, a young male, presented with testicular involvement and without any cutaneous manifestation, which is extremely rare [8]. Two months later the patient presented with leukemia-like symptoms with bone marrow involvement. 

FCM is a useful tool and the cells express CD4, CD43, CD45RA, CD56, CD123, CD303, CD304, TCL1, CLA, CD38, CD99, and CD31 [4,7]. Recently the TCF4 (E2-2) transcription factor represents a faithful diagnostic marker [9]. The immunonegativity of immature cells for CD14, CD15, CD36, CD64, and CD68 excludes the possibility of acute myeloid leukemia with monocytic differentiation. Cytogenetic studies may show genomic abnormalities or a normal karyotype [10].

From a pathologist’s perspective, the immunophenotyping profile should be kept in mind before considering a case of BPDCN as an acute undifferentiated leukemia or acute myeloid leukemia with monocytic differentiation.

## References

[1] Facchetti F, Jones DM, Petrella T (2008). Blastic plasmacytoid dendritic cell neoplasm. In: Swerdlow SH, Campo E, Harris NL, Jaffe ES, Pileri SA, Stein H, Thiele J, Vardiman JW (eds). WHO Classification of Tumours of Haematopoietic and Lymphoid Tissues. 4th ed. Lyon, IARC Press,.

[2] Facchetti F, Petrella T, Pileri SA (2017). Blastic plasmacytoid dendritic cell neoplasm. In: Swerdlow SH, Campo E, Harris NL, Jaffe ES, Pileri SA, Stein H, Thiele J, Arber DA, Hasserjian R, Le Beau M, Orazi A, Siebert R (eds). WHO Classification of Tumours of Haematopoietic and Lymphoid Tissues. Revised 4th edition. Lyon, IARC Press.

[3] Bueno C, Almeida J, Lucio P, Marco J, Garcia R, de Pablos JM, Parreira A, Ramos F, Ruiz-Cabello F, Suarez-Vilela D, San Miguel JF, Orfao A (2004). Incidence and characteristics of CD4(+)/HLA DRhi dendritic cell malignancies. Haematologica.

[4] Julia F, Dalle S, Duru G, Balme B, Vergier B, Ortonne N, Vignon-Pennamen MD, Costes-Martineau V, Lamant L, Dalac S, Delattre C, Déchelotte P, Courville P, Carlotti A, De Muret A, Fraitag S, Levy A, Mitchell A, Petrella T (2014). Blastic plasmacytoid dendritic cell neoplasms: clinico immunohistochemical correlations in a series of 91 patients. Am J Surg Pathol.

[5] Shi Y, Wang E (2014). Blastic plasmacytoid dendritic cell neoplasm: a clinicopathologic review. Arch Pathol Lab Med.

[6] Jegalian AG, Buxbaum NP, Facchetti F, Raffeld M, Pittaluga S, Wayne AS, Jaffe ES (2010). Blastic plasmacytoid dendritic cell neoplasm in children: diagnostic features and clinical implications. Haematologica.

[7] Suzuki R, Nakamura S, Suzumiya J, Ichimura K, Ichikawa M, Ogata K, Kura Y, Aikawa K, Teshima H, Sako M, Kojima H, Nishio M, Yoshino T, Sugimori H, Kawa K, Oshimi K; NK-cell Tumor Studt Group (2005). Blastic natural killer cell lymphoma/leukemia (CD56‑positive blastic tumor): prognostication and categorization according to anatomic sites of involvement. Cancer.

[8] Deng W, Yang M, Kuang F, Liu Y, Zhang H, Cao L, Xie M, Yang L (2017). Blastic plasmacytoid dendritic cell neoplasm in children: a review of two cases. Mol Clin Oncol.

[9] Ceribelli M, Hou ZE, Kelly PN, Huang DW, Wright G, Ganapathi K, Evbuomwan MO, Pittaluga S, Shaffer AL, Marcucci G, Forman SJ, Xiao W, Guha R, Zhang X, Ferrer M, Chaperot L, Plumas J, Jaffe ES, Thomas CJ, Reizis B, Staudt LM (2016). A druggable TCF-4 and BRD4-dependent transcriptional network sustains malignancy in blastic plasmacytoid dendritic cell neoplasm. Cancer Cell.

[10] Leroux D, Mugneret F, Callanan M, Radford-Weiss I, Dastugue N, Feuillard J, Le Mée F, Plessis G, Talmant P, Gachard N, Uettwiller F, Pages MP, Mozziconacci MJ, Eclache V, Sibille C, Avet-Loiseau H, Lafage-Pochitaloff M (2002). CD4+, CD56+ DC2 acute leukemia is characterized by recurrent clonal chromosomal changes affecting 6 major targets: a study of 21 cases by the Groupe Francais de Cytogenetique Hematologique. Blood.

